# Interactions between Cells with Distinct Mutations in c-MYC and Pten in Prostate Cancer

**DOI:** 10.1371/journal.pgen.1000542

**Published:** 2009-07-03

**Authors:** Jongchan Kim, Isam-Eldin A. Eltoum, Meejeon Roh, Jie Wang, Sarki A. Abdulkadir

**Affiliations:** 1 Department of Pathology, Vanderbilt University Medical Center, Nashville, Tennessee, United States of America; 2 Department of Pathology, University of Alabama at Birmingham, Birmingham, Alabama, United States of America; Stanford University School of Medicine, United States of America

## Abstract

In human somatic tumorigenesis, mutations are thought to arise sporadically in individual cells surrounded by unaffected cells. This contrasts with most current transgenic models where mutations are induced synchronously in entire cell populations. Here we have modeled sporadic oncogene activation using a transgenic mouse in which c-MYC is focally activated in prostate luminal epithelial cells. Focal c-MYC expression resulted in mild pathology, but prostate-specific deletion of a single allele of the *Pten* tumor suppressor gene cooperated with c-MYC to induce high grade prostatic intraepithelial neoplasia (HGPIN)/cancer lesions. These lesions were in all cases associated with loss of Pten protein expression from the wild type allele. In the prostates of mice with concurrent homozygous deletion of *Pten* and focal c-MYC activation, double mutant (i.e. *c-MYC+;Pten*-null) cells were of higher grade and proliferated faster than single mutant (*Pten*-null) cells within the same glands. Consequently, double mutant cells outcompeted single mutant cells despite the presence of increased rates of apoptosis in the former. The p53 pathway was activated in *Pten*-deficient prostate cells and tissues, but c-MYC expression shifted the p53 response from senescence to apoptosis by repressing the p53 target gene *p21^Cip1^*. We conclude that c-MYC overexpression and *Pten* deficiency cooperate to promote prostate tumorigenesis, but a p53-dependent apoptotic response may present a barrier to further progression. Our results highlight the utility of inducing mutations focally to model the competitive interactions between cell populations with distinct genetic alterations during tumorigenesis.

## Author Summary

In most human cancers, mutations are thought to arise in a single cell or few cells surrounded by their unaffected neighbors. Expansion of mutant cells can then allow the accumulation of additional mutations. The cell–cell interactions that may occur between mutant and unaffected cells or between cells with distinct mutations during tumorigenesis have not been well studied due to the lack of suitable *in vivo* models. To help fill this gap, we generated and characterized transgenic mice in which the oncogene c-MYC is activated focally in prostate epithelial cells. We have also analyzed mice in which prostate epithelial cells with two mutations (c-MYC overexpression and loss of Pten tumor suppressor) are found next to cells with a single mutation (loss of Pten). Although loss of Pten in the prostate is tumorigenic, it also activates a cellular senescence response which restrains further tumor progression. We found that concurrent c-MYC expression suppressed the senescence response in *Pten*-null cells in favor of apoptosis. *c-MYC*+;*Pten*-null cells proliferated faster than *Pten*-null cells in the same glands, with the net result that *c-MYC*+;*Pten*-null cells outcompete *Pten*-null cells. Our results demonstrate the utility of accurate models to mimic the heterogeneous and incremental nature of human prostate carcinogenesis.

## Introduction

Prevailing models of multistep carcinogenesis posit that oncogenic mutations arise in isolated cells *in situ* followed by clonal expansion. This implies that important competitive interactions occur between mutant and normal cells as well as between cells with distinct oncogenic mutations during tumorigenesis. A detailed understanding of these interactions will further efforts aimed at therapeutic targeting of neoplastic and preneoplastic lesions. However, these interactions have not been well studied *in vivo* due to a paucity of appropriate models. We report here our attempt to model these interactions in a new transgenic model of prostate cancer, focusing on the oncogene c-MYC and the tumor suppressor Pten (Phosphatase and tensin homolog), both of which are implicated in human prostate tumorigenesis [1]. c-MYC overexpression is a common early event in prostate cancer [2],[3] while *PTEN* is deleted/mutated in ∼30% of primary human prostate cancers [4]–[8]. Previous attempts at modeling c-MYC overexpression in the mouse prostate have used prostate-specific promoters that target transgene expression to a majority of the cells in the prostatic epithelium. Depending on the strength of the promoter used, this resulted in various grades of mouse prostatic intraepithelial neoplasia (mPIN) or adenocarcinoma [3],[9]. Similarly, *Pten*-mutant mice develop mPIN and prostate cancer [10]–[13] and *Pten* inactivation can cooperate with mutations in oncogenes and tumor suppressors in prostate tumorigenesis, including *p27^Kip1^* [14],[15], *Trp53* [13] and *Fgf8b* [16].

*Pten* loss has been reported to activate the p53 pathway, leading to senescence [13],[17],[18]. Activation of p53 may lead to cell cycle arrest or apoptosis depending on the downstream target genes induced (i.e. cell cycle arrest genes e.g. *p21^cip1^* versus apoptotic genes e.g. *PUMA*). There is potential cross-talk between the c-MYC and the p53 pathways at various levels depending on the cell context [19]. c-MYC activation can increase ARF expression, thereby stabilizing p53 protein levels [20], and c-MYC can repress expression of some p53 target genes such as *p21^Cip1^* [21]. In addition, Pten and p53 coordinately control c-Myc protein levels with the latter playing a critical role in maintaining the stemness of murine neural stem cells [22].

To model the sporadic genetic alterations that are thought to occur during human somatic tumorigenesis [23], we generated a transgenic mouse in which a latent *c-MYC* transgene can be focally activated in the prostatic epithelium by Cre expression. We have also deleted one or both copies of *Pten* in the prostate concurrently with focal c-MYC overexpression, in order to examine the interactions of cell populations with distinct mutations within the same gland.

## Results

### Generation of *PbCre4;Z-MYC* mice with focal, prostate-specific c-MYC overexpression

**Figure 1:**
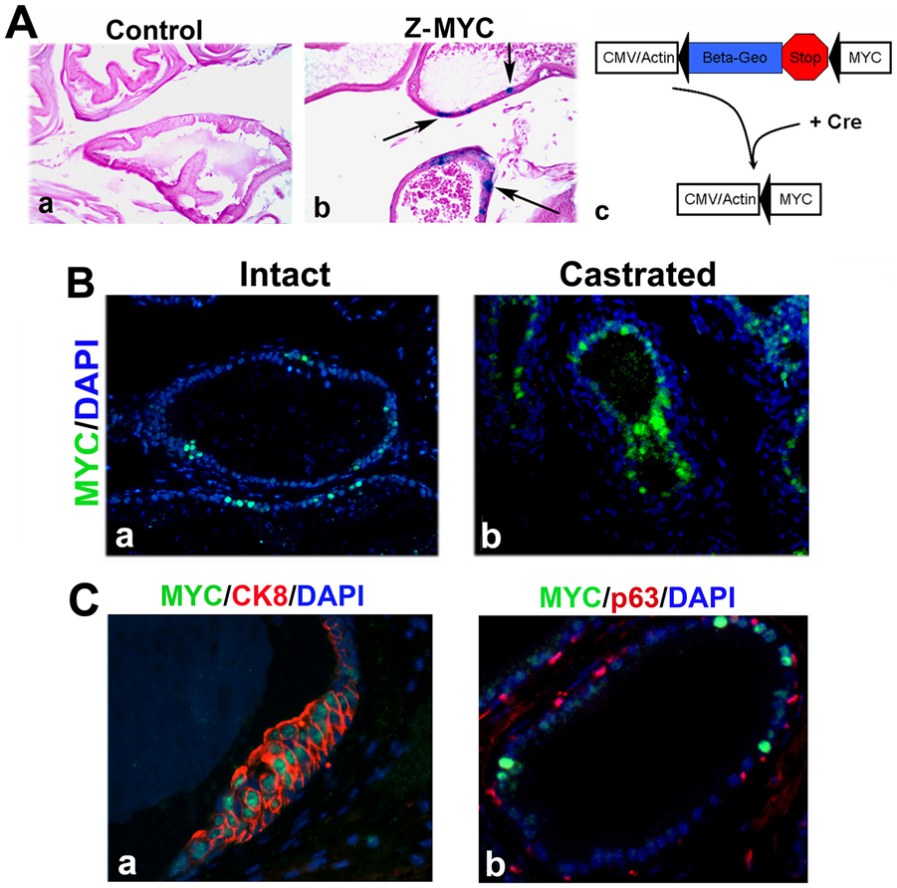
c-MYC expression in *PbCre4;Z-MYC* mouse prostate is mosaic and uncoupled from androgen regulation. (A) LacZ stain depicts mosaic expression of transgene in *Z-MYC* prostate. Arrows indicate LacZ-positive cells. The frequency of transgene-expressing cells varied depending on glands and lobes, but quantitative analysis indicated that overall, LacZ-positive cells comprised ∼17% of the epithelial cells in any LacZ-positive gland (a) and (b). (c) shows schematic representation of Cre excision of the *Z-MYC* construct. c-MYC expression is activated and regulated by *CMV/Actin* promoter after Cre-excision. (B) Immunostaining verifies sporadic c-MYC expression in *PbCre4;Z-MYC* prostate epithelium of intact mouse (a) and 17 weeks after castration (b). (C) c-MYC colocalizes with cytokeratin 8 (CK8) (a) but not with p63 (b) in *PbCre4;Z-MYC* mouse prostates.

To target focal c-MYC expression in the prostate epithelium, we used *Z-MYC* mice that carry a single copy transgene in which the *CMV enhancer/beta actin promoter* drives expression of the *beta-geo* gene and a latent *c-MYC* transgene [24] (Figure 1A). Staining for beta-galactosidase confirmed mosaic expression in the prostate epithelium (Figure 1A). To induce c-MYC expression focally in the prostate, we crossed *Z-MYC* mice to *PbCre4* mice [25] which express Cre recombinase in the prostatic epithelium (Figure 1A). Bigenic *PbCre4;Z-MYC* mice expressed c-MYC focally in cytokeratin 8 (CK8) positive prostate luminal epithelial cells but not p63+ basal cells (Figure 1C). Furthermore, c-MYC expression is not abrogated in castrated animals indicating that the use of the *CMV enhancer/beta actin promoter* in our model has uncoupled prostate-specific expression from androgen-dependent gene regulation (Figure 1B).

### Mild pathology due to focal c-MYC overexpression

**Figure 2:**
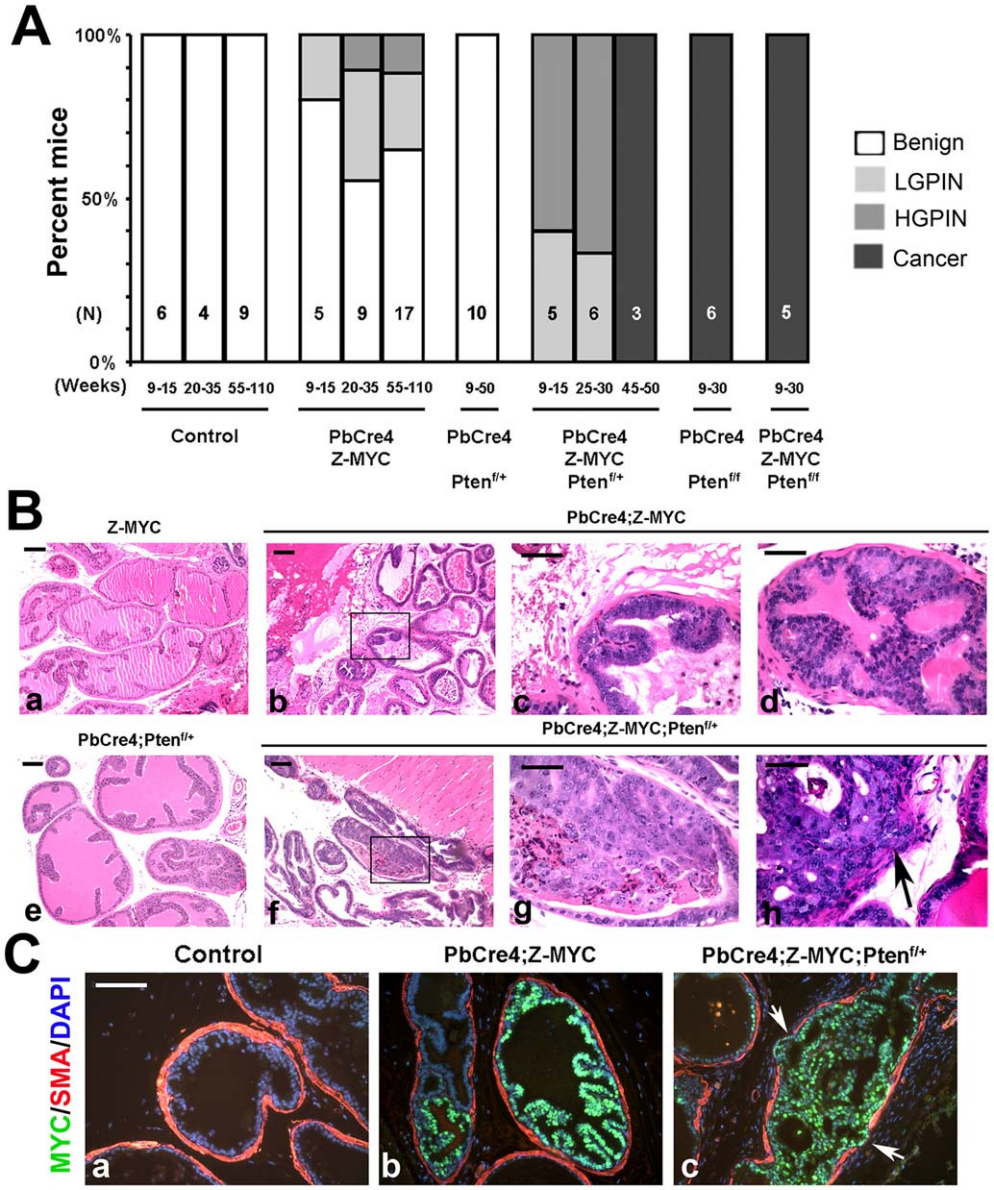
Pathology of *PbCre4;Z-MYC* and compound *c-MYC/Pten* mutant mice. (A) Hematoxylin and eosin (H&E)–stained prostate sections were analyzed and the summary of the pathological grading is shown. (N, number of mice analyzed). (B) Representative images of H&E-stained sections show benign glands in control (a), focal LGPIN in *PbCre4;Z-MYC* (boxed region in b and higher magnification in (c), focal HGPIN lesion in *PbCre4;Z-MYC* (d), benign glands in *PbCre4;Pten^f/+^* (e), focal HGPIN lesions in *PbCre4;Z-MYC;Pten^f/+^* (boxed region in f and higher magnification in g) and focal micro-invasive cancer lesion (arrow) in *PbCre4;Z-MYC;Pten^f/^* ^+^ (h). Scale bars: 100 µm in (a,b,e,f) and 50 µm in (c,d,g,h). (C) c-MYC and smooth muscle actin (SMA) staining. Arrows in (c) indicate focal areas of disruption of SMA (micro-invasion) in *PbCre4;Z-MYC;Pten^f/+^* prostate. Scale bar: 100 µm.

**Figure 3:**
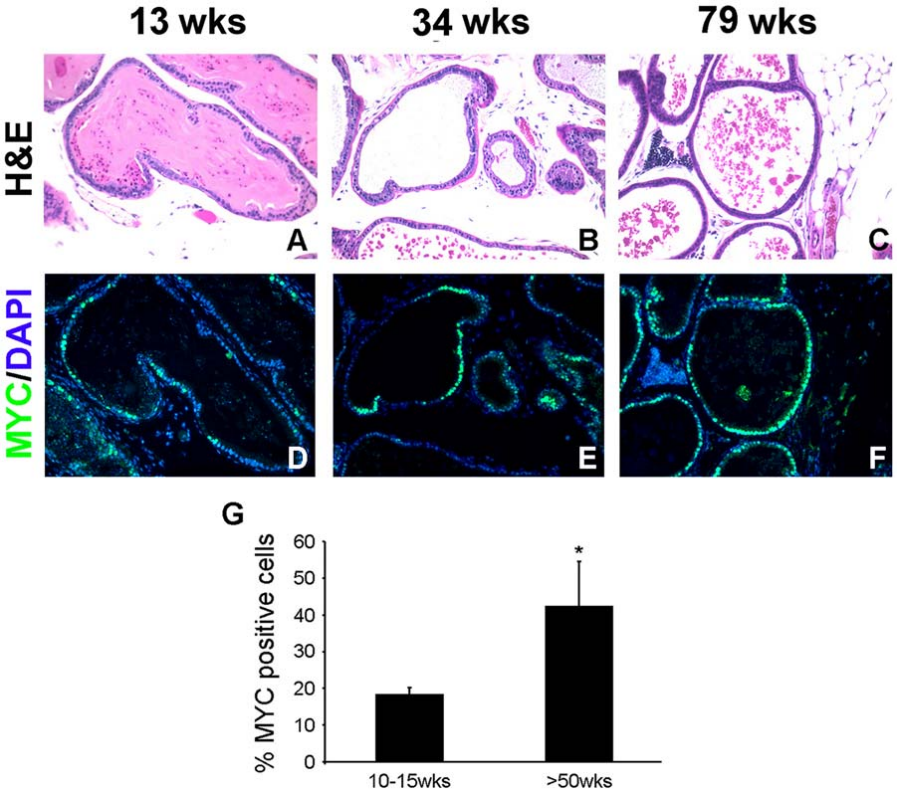
Time-dependent increase in c-MYC–expressing cells without discernible histopathology in a subset of *PbCre4;Z-MYC* mice. (A–C) H&E images demonstrate absence of histopathological abnormalities in *PbCre4;Z-MYC* prostates at various ages. (D–F) Adjacent sections subject to immunofluorescent staining for c-MYC demonstrate the numbers of c-MYC–overexpressing cells increase in a time-dependent manner. (G) The number of c-MYC–positive cells in any c-MYC–positive gland was quantitated based on the immunostaining shown in (D–F). N = 3–4 prostate samples per group. *p<0.05.

Focal c-MYC activation resulted in mild pathology, with most prostates showing normal histology or low grade mPIN (LGPIN) lesions up to 2 years of age (Figure 2A and 2B). This is unlikely to be due to low level c-MYC expression as the *CMV enhancer/beta actin promoter* is known to drive high level transgene expression. A closer examination of the c-MYC expression pattern in the prostates of *PbCre4;Z-MYC* mice with no pathology showed that in young mice, the frequency of c-MYC-positive cells was ∼18% of the epithelial cells in c-MYC-positive glands (Figure 3G), evocative of the frequency of LacZ-positive cells (∼17%) in *Z-MYC* prostates (Figure 1A). By 1 year, the frequency of c-MYC positive cells has increased to ∼43% (Figure 3G). The lack of discernible histological abnormality in the prostates of a large fraction of older *PbCre4;Z-MYC* mice in the face of abundant c-MYC expression is reminiscent of the phenomenon of “field cancerization” in human tumorigenesis where incipient mutant cells occupy tissue fields without any apparent pathology (Figure 3A–3F) [26]. These histologically normal but mutant cells may serve as targets for transformation with additional genetic mutations.

### Focal c-MYC expression cooperates with *Pten* heterozygosity

**Figure 4:**
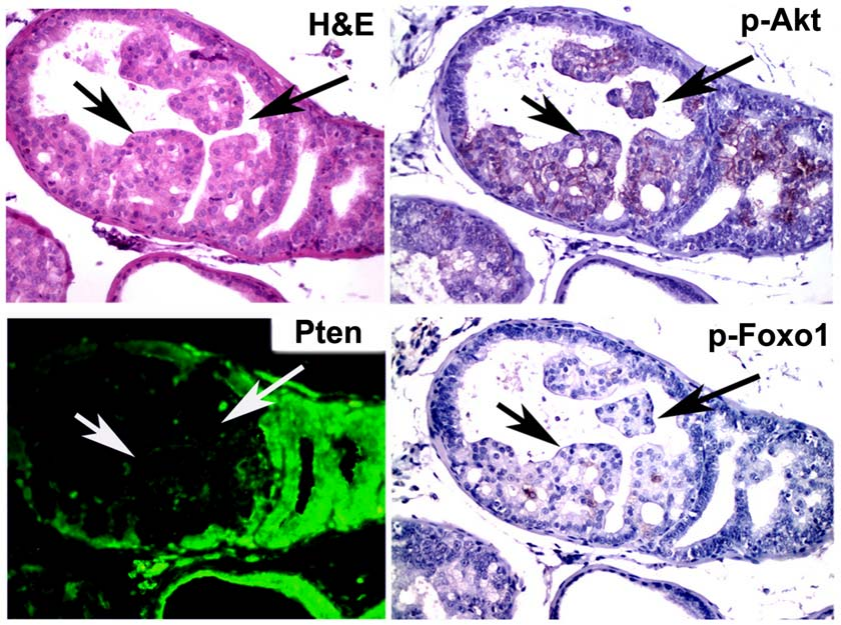
Focal loss of Pten expression in PIN lesions of *PbCre4;Z-MYC;Pten^f/+^* mouse prostate. In focal PIN lesion (arrows), Pten protein expression is lost and phosphorylation of Akt and Foxo1 increased.

Next, we generated compound mutant mice with prostate-specific deletion of one or both alleles of *Pten* concurrently with focal activation of c-MYC. Examination of *PbCre4;Z-MYC;Pten^f/+^* prostates revealed clear cooperation between c-MYC overexpression and *Pten* heterozygosity (Figure 2). As reported previously [10],[11] and confirmed by us here, conditional deletion of a single *Pten* allele had little effect on the prostate with mice up to 50 weeks of age showing minimal abnormalities (Figure 2A and 2B). By ∼10 weeks of age however, *PbCre4;Z-MYC;Pten^f/+^* mice already have evidence of focal HGPIN lesions. Over time, these animals develop micro-invasive cancer as confirmed by the presence of areas with disruption in smooth muscle actin (SMA) immunoreactivity (Figure 2B and 2C). We used immunohistochemistry to examine the status of the wild type *Pten* allele in the HGPIN/cancer lesions in *PbCre4;Z-MYC;Pten^f/+^* mice. Consistently, all lesions examined (N = 8 mice) showed loss of Pten protein expression and phosphorylation of its downstream signaling components Akt and Foxo1 [27] (Figure 4).

### *c-MYC+;Pten*-null cells outcompete Pten-*null* cells in the same glands

**Figure 5:**
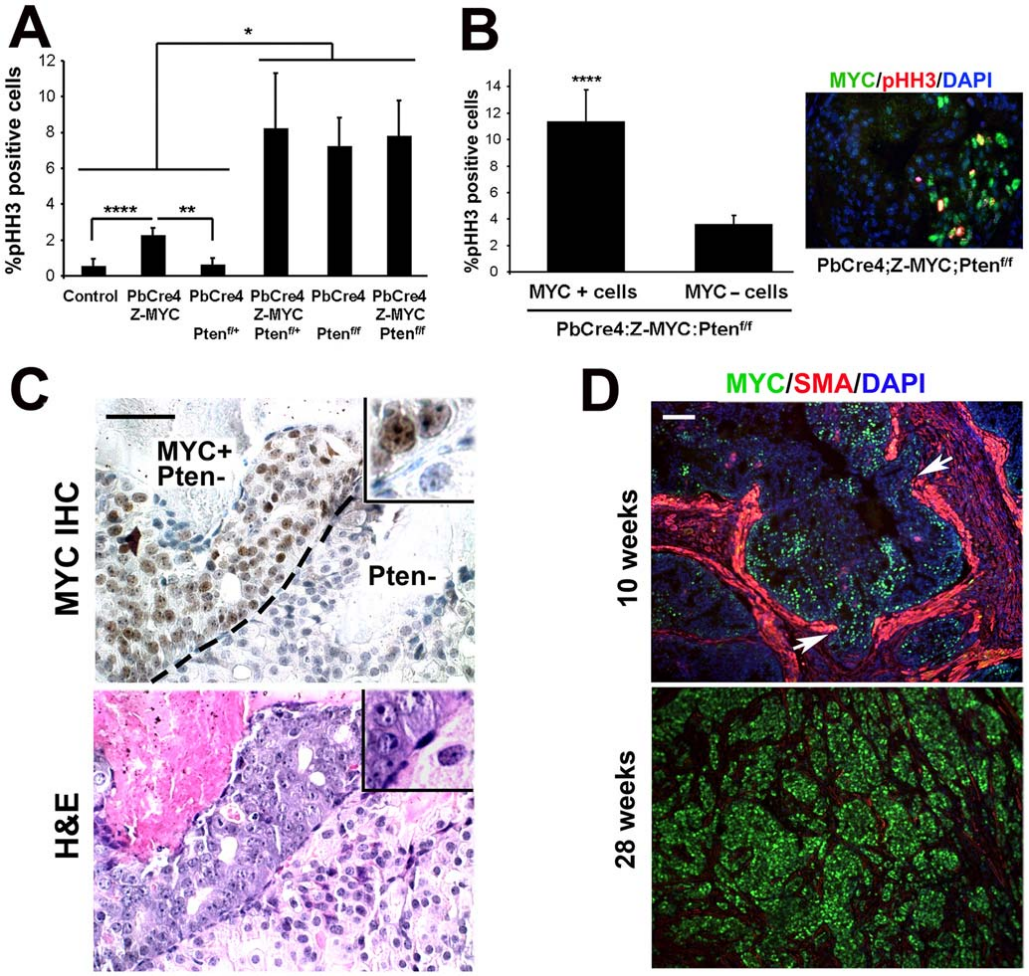
c-MYC expression increases the proliferation and tumorigenicity of *Pten*-deficient cells. (A) Proliferation was determined by analysis of phospho-histone H3 staining. (B) Phospho-histone H3 index in c-MYC-positive or c-MYC-negative cells in *PbCre4;Z-MYC;Pten^f/f^* prostates. Inset: double staining shows colocalization of phospho-histone H3 with c-MYC in *PbCre4;Z-MYC;Pten^f/f^* prostate. N = 3–4 mice per group. *p<0.05, **p<0.005, ****p<0.01. (C) c-MYC staining identifies MYC-expressing cells next to MYC-negative cells in the same gland of *PbCre4;Z-MYC;Pten^f/f^* mouse prostate. An adjacent H&E-stained section is also shown. Note distinct, higher grade pathology of c-MYC+ cells. Scale bar: 50 µm. (D) *c-MYC+;Pten*-null cells outcompete *Pten*-null cells. Prostates from 10-week-old and 28-week-old *PbCre4;Z-MYC;Pten^f/f^* mice were stained for c-MYC and smooth muscle actin. At 10 weeks, c-MYC expression is focal; at 28 weeks, it is uniform. Arrows indicate discontinuity of smooth muscle actin (focal micro-invasion). Scale bar: 100 µm.

We analyzed proliferation by staining for phospho-histone H3 (pHH3), a mitotic marker. Proliferation was increased significantly in *PbCre4;Z-MYC* prostates relative to controls, and *Pten* heterozygosity synergistically increased it further (Figure 5A). The proliferation rates in *PbCre4;Pten^f/f^* and *PbCre4;Z-MYC;Pten^f/f^* were similarly elevated. However, the focal nature of c-MYC expression in our model means that analysis of total proliferation in the *PbCre4;Z-MYC;Pten^f/f^* prostates may not be an accurate measure of the proliferation in foci of c-*MYC+;Pten*-null cells. To overcome this, we performed double staining for c-MYC and phospho-histone H3. As shown in Figure 5B, double mutant (*c-MYC+;Pten*-null) cells were more proliferative than single mutant (*Pten*-null) cells within the same prostate glands. Furthermore, double mutant cells are histologically distinct from adjacent single mutant cells. The double mutant cells are of higher pathological grade with larger nuclei, high nuclear∶cytoplasmic ratios, hyperchromatic nuclei with prominent chromocenters, focal chromatin clearing and prominent single or sometimes multiple nucleoli (Figure 5C and Figure S1). In addition, apoptotic and mitotic figures are prominent. Single mutant (*Pten*-null) cells on the other hand showed low nuclear grade with comparatively small and uniform nuclei, abundant pale cytoplasm and low nuclear∶cytoplasmic ratios. These cells also have inconspicuous nucleoli and the chromatin is comparatively fine (Figure 5C). These observations suggest that *c-MYC+;Pten*-null cells may out-compete *Pten*-null cells within the same prostate gland over time. Indeed, analysis of *PbCre4;Z-MYC;Pten^f/f^* animals showed that at early ages, c-MYC expression was focal within glands, but in older mice, lesions show uniform c-MYC expression, suggesting clonal expansion of c-MYC-positive cells in a time-dependent manner (Figure 5D).

### Pten loss does not protect c-MYC overexpressing prostate cells from apoptosis

**Figure 6:**
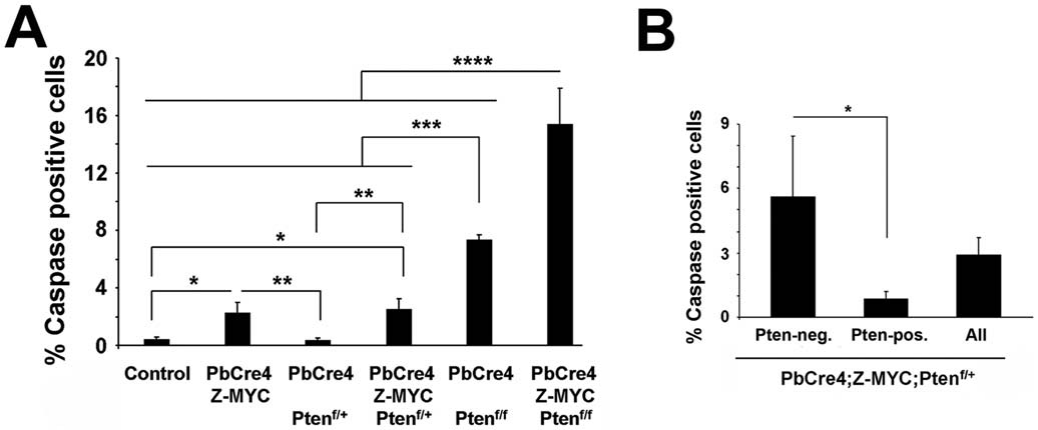
Analysis of apoptosis in *c-MYC/Pten* compound mutant mice. (A) Apoptosis was analyzed by staining for activated Caspase 3. (B) Sections from *PbCre4;Z-MYC;Pten^f/+^* prostates were doubly stained for activated Caspase 3 and Pten, and the number of apoptotic cells was quantitated in Pten-positive and Pten-negative epithelial cells. N = 3–4 mice per group. *p<0.05, **p<0.005, ***p<0.001 and ****p<0.01.

Analysis of apoptosis by staining for activated Caspase 3 shows that control and *PbCre4;Pten^f/+^* prostates had low levels of apoptosis, consistent with their normal histology, while focal expression of c-MYC in *PbCre4;Z-MYC* prostates modestly increased apoptosis (Figure 6A). Although c-MYC overexpression is known to induce apoptosis in several tissues, this depends on many variables including the level of c-MYC overexpression and the “tissue context” [28],[29]. The levels of apoptosis seen in *PbCre4;Z-MYC* prostates are consistent with increased cell turnover due to enhanced proliferation. *Pten*-null prostates also had increased rates of apoptosis, and c-MYC overexpression further enhanced this effect (Figure 6A). These results were surprising as Pten loss is known to activate pro-survival pathways. Therefore, we sought to determine if apoptosis is increased in HGPIN/cancer cells that have lost Pten expression in our *PbCre4;Z-MYC;Pten^f/+^* mice. Double staining for Pten and activated Caspase 3 and quantitative analysis indicated higher rates of apoptosis in Pten-negative cells compared to Pten-positive cells (Figure 6B). Thus Pten loss does not protect prostate cells from apoptosis due to c-MYC overexpression.

In addition to Akt, the c-Jun N-terminal kinase (Jnk) pathway is known to be activated in *Pten*-deficient cells and tumors [30],[31]. We confirmed that the Jnk pathway is activated in both *Pten*-null and c-MYC-overexpressing/*Pten*-null prostates by immunohistochemistry for phospho-Jnk (Figure S2A). Since Jnk is well known to have the ability to activate apoptosis, cell survival and proliferation depending on cellular signal stimuli and cellular contexts [32], we asked if increased Jnk activity sensitizes *Pten*-deficient cells to apoptosis. We used small hairpin RNA to stably downregulate PTEN in the benign human prostatic cell line RWPE1. However, treatment with the Jnk inhibitor (SP600125) led to an increase in apoptosis in PTEN knockdown cells in a dose-dependent manner, suggesting that PTEN loss-induced Jnk activity is anti-apoptotic, rather than pro-apoptotic (Figure S2).

### c-MYC shifts the p53 response in *Pten*-deficient prostate cells from senescence to apoptosis

**Figure 7:**
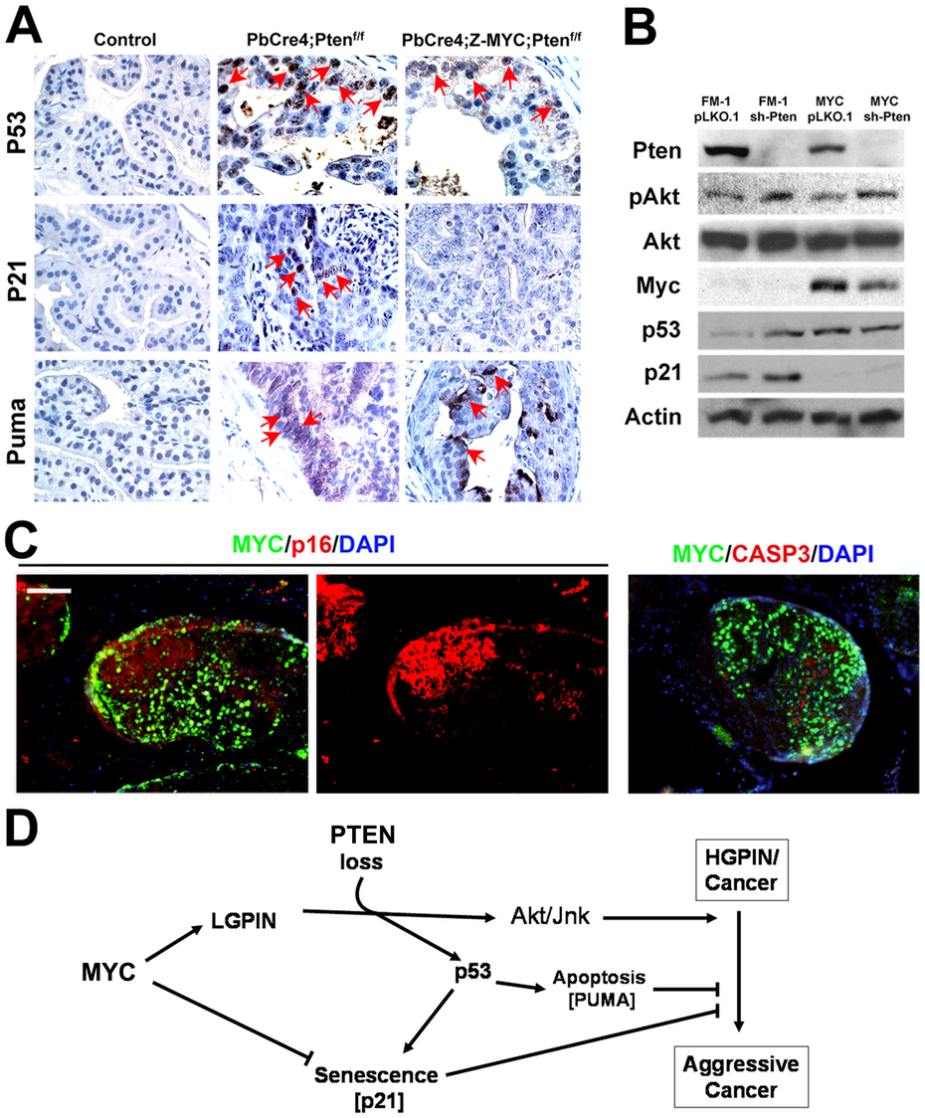
c-MYC and p53 pathway status in *c-MYC/Pten* mutant prostate cells. (A) Immunohistochemistry for p53, p21 and Puma. Prostate sections from mice of the indicated genotypes were stained with the indicated antibodies and nuclei were counterstained with hematoxylin. Positive cells (brown) are indicated by red arrows. (B) Western blots show expression of the indicated proteins in RWPE-1 cells expressing c-MYC, PTEN knockdown, or control vectors (FM-1, pLKO.1). PTEN and phospho-Akt (pAkt) blots confirm efficiency of PTEN knockdown. (C) Double immunofluorescent stains for c-MYC/p16 and c-MYC/activated Caspase 3 in *PbCre4;Z-MYC;Pten^f/f^* prostates. Scale bar: 100 µm. (D) Model of interactions between c-MYC and Pten with the p53 pathway in c-MYC–initiated prostate cancer. Focal c-MYC overexpression leads to LGPIN and facilitates loss of Pten leading to HGPIN/invasive cancer. Activation of the p53 pathway due to Pten loss could lead to senescence or apoptosis. c-MYC expression is proposed to shift this response towards apoptosis by repressing p21 expression.

It is known that *Pten* loss can activate the p53 pathway in the prostate cells and activation of the p53 pathway could lead to either senescence or apoptosis depending on the particular p53 target genes induced [13],[17],[18],[33]. We therefore sought to examine activation of the p53 pathway in our *c-MYC/Pten* model and to determine whether concurrent c-MYC expression alters the p53 response. We observed induction of p53, its targets p21^cip1^ and PUMA in *Pten*-null prostates (Figure 7A). However, while p53 and PUMA were induced in c-MYC-overexpressing *Pten*-null prostates, p21^cip1^ expression was not (Figure 7A), consistent with the notion that c-MYC represses p21^cip1^ expression [21]. Similar results were obtained in RWPE-1 cells (Figure 7B). While p53 and p21^cip1^ were induced upon PTEN knockdown, c-MYC overexpression repressed p21^cip1^ expression (Figure 7B). We hypothesized that in *Pten*-deficient cells with activation of the p53 pathway, repression of p21^cip1^ by c-MYC may switch the senescent response to apoptosis. Indeed, using immunofluorescence, we found that in *PbCre4;Z-MYC;Pten^f/f^* prostates, p16^ink4a^ expression (a marker of senescence) is mainly localized to c-MYC-negative cells while apoptosis (activated Caspase 3) is found predominantly among c-MYC-positive cells (Figure 7C). Thus *Pten*-deficiency activates the p53/p21^cip1^ pathway but concurrent c-MYC overexpression shifts the output of the pathway from senescence to apoptosis at least partly by repressing p21^cip1^.

## Discussion

Human prostate carcinogenesis is focal, random, and incremental, but current mouse models do not faithfully recapitulate this. Consequently, the competitive/cooperative interactions that may occur between mutant and normal cells during the early stages of tumorigenesis have not been well studied. The model described here exploits the stochastic expression of a “Cre-activatable” c-MYC transgene (*Z-MYC*) to induce c-MYC expression in isolated cells surrounded by normal cells. As illustrated by our studies when the *Z-MYC* mouse is crossed with prostate-specific *Pten* deletion, the focal nature of c-MYC expression allows analysis of cell populations with different genetic alterations within the same prostate gland.

Our studies have yielded several insights. First, focal expression of c-MYC in prostate luminal epithelial cells, even though driven by the *CMV enhancer/beta actin* promoter, results in remarkably mild pathology with many mice showing histologically normal prostates and a subset of mice demonstrating LGPIN lesions. These results imply a remarkable tolerance of luminal epithelial cells to c-MYC expression. We showed that the acquisition of additional genetic mutations is essential for the appearance of discernable pathology by the fact that introduction of *Pten* heterozygosity into these animals resulted in cooperativity, with the development of HGPIN/cancer lesions which in all cases were associated with loss of Pten protein expression from the wild type allele. These observations highlight an important point about c-MYC-expressing cells in histologically “normal” glands, as may occur in tumors and tissues with “field cancerization” [26], [34]–[37] in that the overexpression of c-MYC in histologically “normal” cells may facilitate the acquisition of secondary mutations. Although it remains to be established whether loss of Pten expression is due to genetic, epigenetic or post-transcriptional control, c-MYC expression may facilitate acquisition of secondary mutations by increasing cell turnover and/or genomic instability [38],[39].

Our *PbCre4;Z-MYC;Pten^f/f^* mice allowed us to examine the behavior of prostate cells with distinct mutations in the same prostate. c-MYC expression clearly confers an additional proliferative advantage to *Pten*-null prostate cells, allowing *c-MYC+;Pten*-null cells to outcompete *Pten*-null cells. However, *Pten* deficiency did not alleviate apoptosis in *c-MYC+;Pten*-null cells. This may appear surprising in light of the well-known, pro-survival effect of Pten loss [40] and a report that Pten loss decreased the apoptosis engendered by the inactivation of retinoblastoma (pRb) family proteins by a truncated SV40 T large antigen in the mouse prostate [41]. Nevertheless, previous studies of mice with conditional deletion of *Pten* in the prostate and testicular germline cells have noted an increased rate of apoptosis upon *Pten* deletion [10],[11],[42] and Radziszewska *et al* recently showed that deleting *Pten* concurrently with c-MYC activation in pancreatic beta cells led to increased apoptosis [43]. Furthermore, *Pten* deficiency has been reported to activate the p53 pathway leading to senescence [13],[18],[44] as well as to sensitize cells to ROS-induced apoptosis [17].

Based on our studies and published reports, we propose the following model of cooperativity between c-MYC and Pten in prostate cancer (Figure 7D): Overexpression of c-MYC initiates tumorigenesis by facilitating loss of *Pten*. The latter leads to the activation of the p53 pathway, which can result in either senescence or apoptosis depending on the predominant *Trp53* target genes induced (i.e. cell cycle arrest genes e.g. *p21^cip1^* versus pro-apoptotic genes e.g. *PUMA, Bax* etc.). The expression of c-MYC drives cells down the apoptotic pathway as it selectively represses the cell cycle arrest-inducing target gene *p21^cip1^*.

To summarize, we report a new Cre-dependent prostate cancer mouse model that reflects the focal, random and incremental nature of human prostate carcinogenesis. We show that focal c-MYC expression cooperates with *Pten* heterozygosity to promote tumor progression due to the selection of cells with loss of Pten expression. In addition, cells mutant for both *c-MYC* and *Pten* outcompete single *Pten*-mutant cells within the same prostates although *Pten*-deficiency sensitizes cells to apoptosis that is associated with activation of the p53 pathway and exacerbated by c-MYC expression. Our results highlight the utility of modeling focal oncogene activation to study the interactions between cell populations with different genetic alterations in tumorigenesis.

## Materials and Methods

### Animals

*Z-MYC*, *PBCre4* and *Pten^f/f^* mice have been described [24],[25],[45]. Female *Z-MYC* mice (B6/129) were bred to male *PbCre4* mice (B6) obtained from MMHCC, Frederick, to generate *PbCre4;Z-MYC* offspring and littermate controls. *Pten^f/f^* mice (B6/129) were obtained from The Jackson Laboratory. To generate compound mutant mice, we generated *PbCre4;Pten^f/+^* males and *Z-MYC;Pten^f/+^* females which were further bred to obtain *PbCre4;Z-MYC*, *PbCre4;Pten^f/f^*, *PbCre4;Z-MYC;Pten^f/+^* and *PbCre4;Z-MYC;Pten^f/f^* offspring for experiments as well as their littermate controls. Animal care and experiments were carried out according to the protocols approved by the Institutional Animal Care and Use Committees at Vanderbilt University.

### LacZ stain

Beta-galactosidase staining followed by counterstaining with nuclear fast red was performed as described [24].

### Histology and immunohistochemistry

Tissues were prepared for histopathological analysis as described [46], and slides were reviewed by IEA based on published criteria [47]. Immunohistochemical analyses were performed as described [46]. The following antibodies were used, in some cases with Tyramide Signal Amplification (TSA; Perkin Elmer): anti-activated Caspase 3 (rabbit, 1∶500, Cell Signaling), anti-phospho-histone H3 (rabbit, 1∶500, Upstate), anti-phospho-Akt (rabbit, 1∶100, Cell Signaling), anti-phospho-Foxo1 (rabbit, 1∶50, Santa Cruz), anti-c-MYC (rabbit, 1∶15,000 with TSA, Santa Cruz), anti-Pten (rabbit, 1∶200 with TSA, Cell Signaling), anti-cytokeratin 8 (mouse, 1∶2000, Sigma), anti-p63 (PIN Cocktail, Biocare Medical), anti-p53 (rabbit, 1∶5000 with TSA, Santa Cruz), anti-p21 (mouse, 1∶50, Santa Cruz), anti-smooth muscle actin (mouse, 1∶2000, Sigma), anti-p16 (rabbit, 1∶1000, Santa Cruz), anti-Puma (rabbit, 1∶200, Cell Signaling) and anti-phospho-Jnk antibody (rabbit, 1∶100, Cell Signaling). For double immunofluorescenct stains, c-MYC or Pten detected by 1^st^ primary antibodies were amplified by TSA system (green, Fluorescein). Alexa Fluor 594 (red)-labeled 2^nd^ secondary antibodies (Molecular Probes) were used to detect 2^nd^ primary antibodies (anti-cytokeratin 8, anti-p63, anti-smooth muscle actin, anti-phospho-histone H3, anti-p16 and anti-activated Caspase 3). Nuclear stain (DAPI) and sample mounting were performed using Vectashield mounting medium (Vector Laboratories).

### Proliferation and apoptosis assay

At least 500 cells per sample were counted and quantitated after immunohistochemistry for phospho-Histone H3 and activated Caspase 3, respectively. N = 3–4 prostate samples from 9–15 week-old mice per group.

### Cell lines

RWPE-1, benign human prostate epithelial cell line (ATCC) was cultured in keratinocyte serum-free media supplemented with bovine pituitary extract and EGF (Invitrogen). We used lentiviral-mediated gene transfer to generate PTEN knockdown/c-MYC overexpressing cells. 293FT packaging cells were plated on 10 cm culture dishes and transfected with PTEN shRNA construct/pLKO.1 vector control (Sigma) or the c-MYC construct/FM-1 vector control along with vesicular stomatitis virus glycoprotein (VSVG) envelope plasmid and delta 8.9 packaging plasmid to produce lentivirus. The FM-1 vector was obtained from J. Milbrandt [48] and was used to clone in human c-MYC cDNA. Three days after transfection, medium containing viral particles was collected and added to RWPE-1 for infection with polybrene (8 µg/ml). 24 hours post infection, medium was changed and another 24 hours later puromycin (1 µg/ml) was added for selection of sh-Pten/pLKO.1 cells. YFP-positive c-MYC/FM-1 cells were sorted by flow cytometry.

### Western blot analyses

These were performed as described [49] using the following antibodies: anti-Pten (mouse, 1∶1000, Cell signaling), anti-phospho-Akt (rabbit, 1∶2000, Cell signaling), anti-total Akt (rabbit, 1∶2000, Cell signaling), anti-c-MYC (mouse, 1∶500, Santa Cruz), anti-p53 (mouse, 1∶1000, Santa Cruz), anti-p21(mouse, 1∶1000, Santa Cruz) and anti-beta-actin antibody (goat, 1∶1000, Santa Cruz).

### Jnk inhibitor treatment and immunocytochemistry for activated Caspase 3

Coverslips were placed on the 24-well plates and 300,000 control or Pten knockdown RWPE-1 cells were plated on the coverslips. Next day, cells were treated with Jnk inhibitor (SP600125) or vehicle (DMSO) at 0, 10 or 50 µM for one hour. Then cells were washed with phosphate buffered saline and supplement-free medium was added to induce apoptosis. After 48 hours, immunocytochemistry for activated Caspase 3 was performed and apoptosis was quantitated from triplicate data per group.

### Statistical analyses

We compared groups by using *t*-test. Values are considered statistically significant at *P*<0.05. Quantitative variables are expressed as means±SD while categorical variables are expressed as numbers (%).

## Supporting Information

Figure S1 Histopathology of *PbCre4;Pten^f/f^* and *PbCre4;Z-MYC;Pten^f/f^* tumors at different ages. *PbCre4;Z-MYC;Pten^f/f^* mice show higher grade lesions. Scale bars: 100 µm and 50 µm in insets: higher magnifications.
(6.19 MB TIF)

Figure S2 Jnk is activated in Pten-deficient cells and is anti-apoptotic. (A) Phospho-Jnk expression is apparent in *PbCre4;Pten^f/f^* and *PbCre4;Z-MYC;Pten^f/f^* prostates (brown). (B) Apoptosis increases in Pten-knockdown RWPE-1 cells when treated with Jnk inhibitor (SP600125). (C) Western blots show that Pten knockdown is efficient in PTEN-shRNA-infected RWPE-1 cells. Immunofluorescence images represent increased apoptosis in PTEN-knockdown cells (red, activated Caspase 3). Nuclei were stained blue. *p<0.01.
(6.79 MB TIF)
