# Supplementary figures and images for: Interactions between Cells with Distinct Mutations in c-MYC and Pten in Prostate Cancer
Source: PLoS Genet. 2009 Jul 3;5(7):e1000542. doi: 10.1371/journal.pgen.1000542 (PMC2697385; doi:10.1371/journal.pgen.1000542)

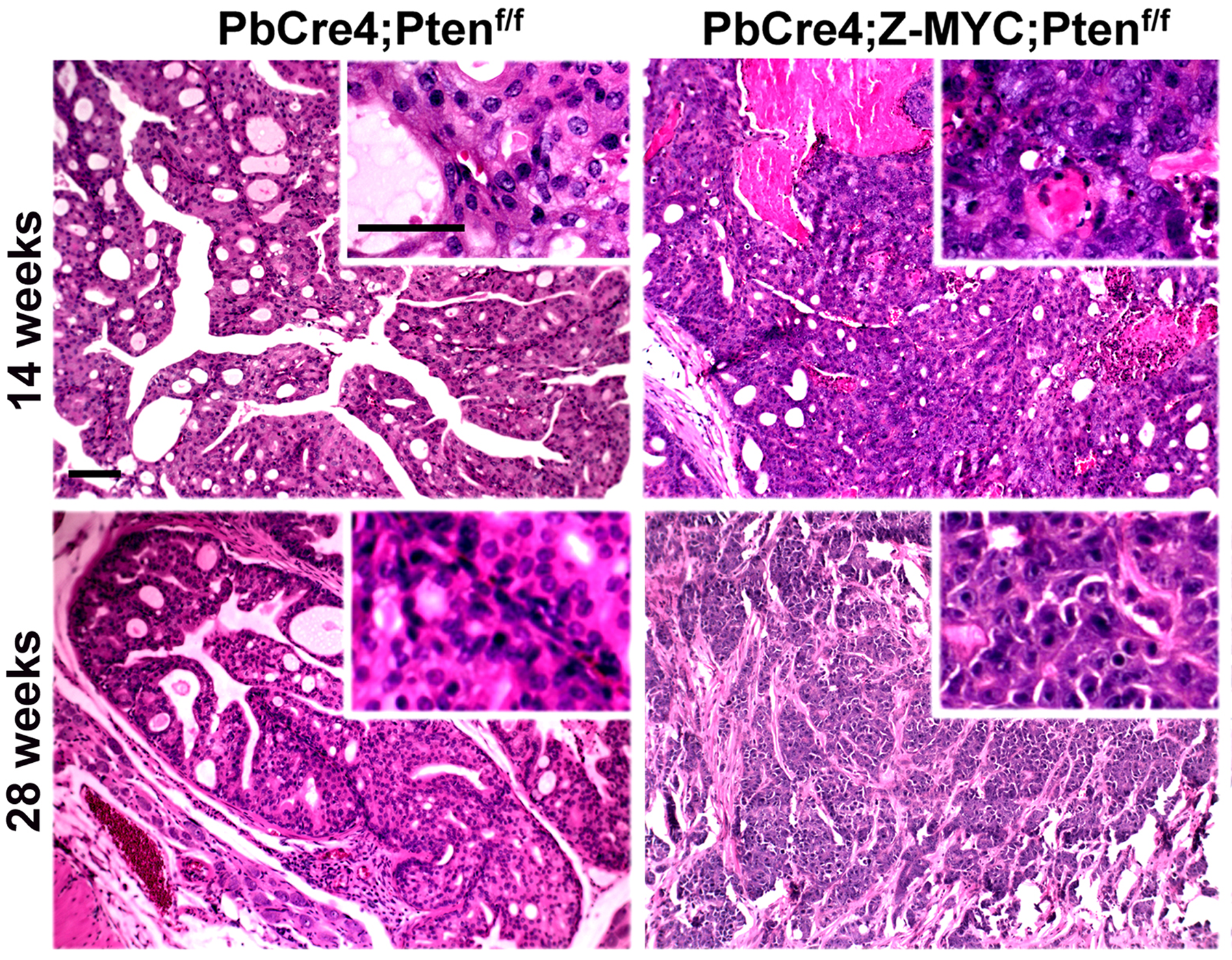

Supplement: Figure S1 — Histopathology of PbCre4;Ptenf/f and PbCre4;Z-MYC;Ptenf/f tumors at different ages. PbCre4;Z-MYC;Ptenf/f mice show higher grade lesions. Scale bars: 100 µm and 50 µm in insets: higher magnifications. (6.19 MB TIF) [file pgen.1000542.s001.tif]

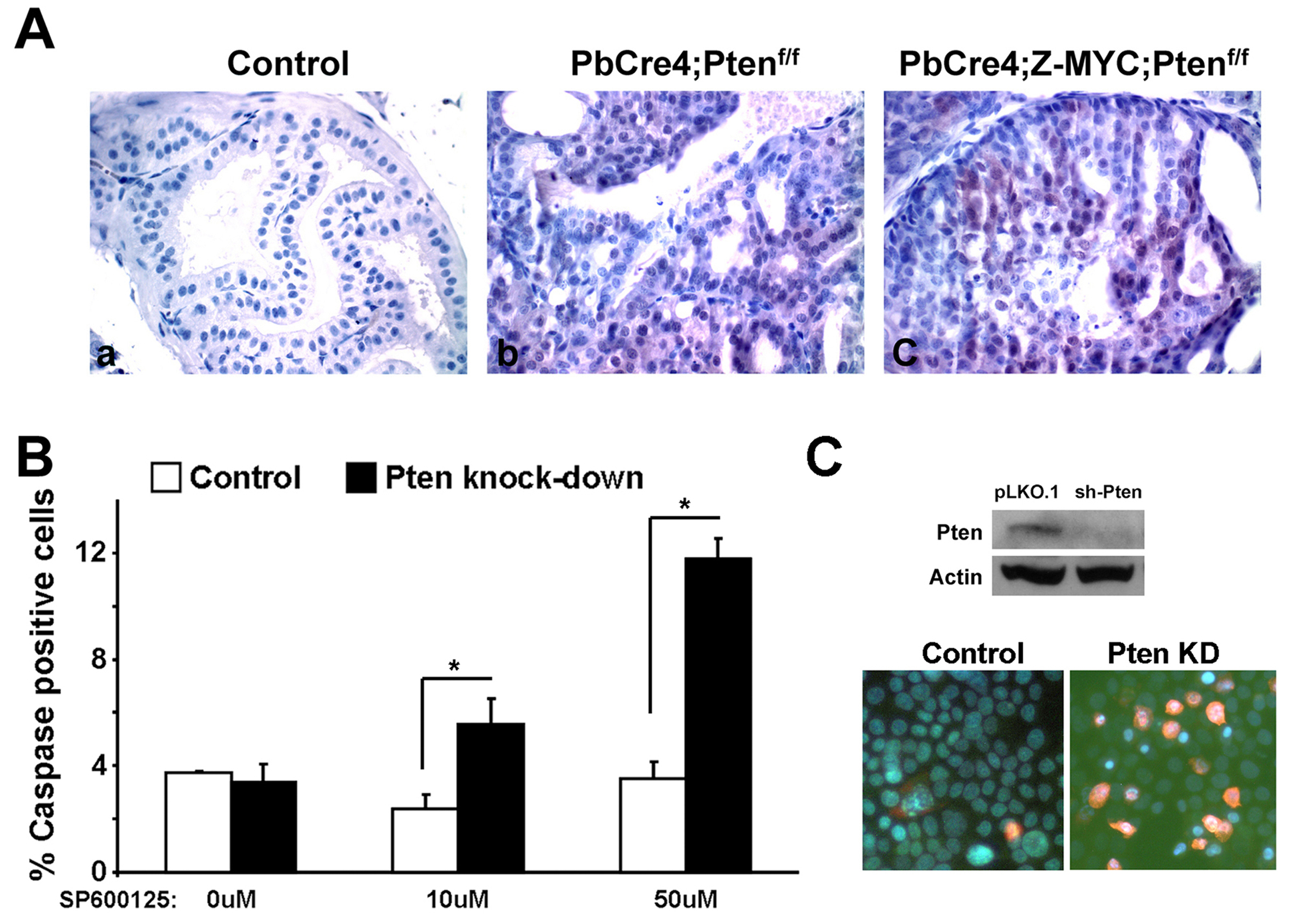

Supplement: Figure S2 — Jnk is activated in Pten-deficient cells and is anti-apoptotic. (A) Phospho-Jnk expression is apparent in PbCre4;Ptenf/f and PbCre4;Z-MYC;Ptenf/f prostates (brown). (B) Apoptosis increases in Pten-knockdown RWPE-1 cells when treated with Jnk inhibitor (SP600125). (C) Western blots show that Pten knockdown is efficient in PTEN-shRNA-infected RWPE-1 cells. Immunofluorescence images represent increased apoptosis in PTEN-knockdown cells (red, activated Caspase 3). Nuclei were stained blue. *p<0.01. (6.79 MB TIF) [file pgen.1000542.s002.tif]
